# Automatic Detection of Attention Shifts in Infancy: Eye Tracking in the Fixation Shift Paradigm

**DOI:** 10.1371/journal.pone.0142505

**Published:** 2015-12-01

**Authors:** Louisa Kulke, Janette Atkinson, Oliver Braddick

**Affiliations:** 1 Division of Psychology and Language Sciences, Faculty of Brain Sciences, University College London, London, United Kingdom; 2 Department of Experimental Psychology, Oxford University, Oxford, United Kingdom; Medical Photonics Research Center. Hamamatsu University School of Medicine, JAPAN

## Abstract

This study measured changes in switches of attention between 1 and 9 months of age in 67 typically developing infants. Remote eye-tracking (Tobii X120) was used to measure saccadic latencies, related to switches of fixation, as a measure of shifts of attention, from a central stimulus to a peripheral visual target, measured in the Fixation Shift Paradigm. Fixation shifts occur later if the central fixation stimulus stays visible when the peripheral target appears (competition condition), than if the central stimulus disappears as the peripheral target appears (non-competition condition). This difference decreases with age. Our results show significantly faster disengagement in infants over 4 months than in the younger group, and provide more precise measures of fixation shifts, than behavioural observation with the same paradigm. Reduced saccadic latencies in the course of a test session indicate a novel learning effect. The Fixation Shift Paradigm combined with remote eye-tracking measures showed improved temporal and spatial accuracy compared to direct observation by a trained observer, and allowed an increased number of trials in a short testing time. This makes it an infant-friendly non-invasive procedure, involving minimal observational training, suitable for use in future studies of clinical populations to detect early attentional abnormalities in the first few months of life.

## Introduction

The ability to shift attention from one visual stimulus to another represents a crucial aspect of human development, allowing infants to direct processing and learning to relevant events, and serving as the basis for developing adult mechanisms of selective attention. Early detection of deficits in shifts of attention is important for the possibility of effective early intervention.

Two well-established behavioural methods, using shifts of gaze to examine attention in young infants, are the Fixation Shift Paradigm—FSP [1–3,review: 4,5], and the gap paradigm [6–11]. In the FSP task infants are initially shown one stimulus centred on a screen. When the infant fixates this central stimulus, the tester initiates the appearance of a second target in the periphery. The central stimulus either remains visible (competition condition), or disappears at the onset of the peripheral target (non-competition condition). In the gap condition there is a time interval between the central stimulus disappearing and the peripheral target appearing [3,7,8]. The time until the first saccade towards the peripheral target provides a measure of infants’ ability to switch attention.

Saccade latencies have been found to differ between conditions, being shorter when there is a time delay between the central target disappearing and the peripheral target appearing and highest in the competition or overlap condition [e.g. 3,8]. Young infants in the first 2–4 months of life show particularly marked long latencies, or do not shift their gaze off the central target at all (sticky fixation) in the competition condition [1–3,9,12]. In ‘sticky fixations’ the infant appears unable to disengage their attention from the central stimulus, although this rarely occurs after age 2–3 months in typical development [e.g. 1,9,13].

A further early improvement in infants’ ability to shift attention is in saccade accuracy. Aslin and Salapatek [14] found that saccades towards peripheral targets were often hypometric within the first two months of life; infants tended to undershoot the peripheral target position, followed by corrective saccades to fixate the target.

In summary, infants’ performance to shift attention, especially under competition, improves with age. Switching attention under competition involves disengaging from the current stimulus, followed by a shift of attention, monitored as an eye-movement to the new target. Infants with one cerebral hemisphere removed due to severe epilepsy (hemispherectomy) can make fixation shifts under non-competition to both sides, but not under competition, to a target appearing in the half-field corresponding to the surgically removed hemisphere [15]. This suggests that automatic engagement with a target only involves subcortical pathways, whereas cortical control becomes necessary for disengagement from a current central stimulus [cf. 3,13,15]. Hence, the developmental improvement in competition conditions is taken as indirect evidence that in the first months of life, attention shifts reflect activity in subcortical networks involving the superior colliculus, with cortical control emerging at around 3 months of age, allowing the infant to shift attention more flexibly at older ages [e.g. 4,16,17].

Poor disengagement under competition, possibly indicating delayed or abnormal cortical maturation, has been found in children with Williams syndrome [18], pre-term born infants [19,20], infants with perinatal insults–HIE or focal lesions [21], siblings of autistic children [11] and infants who had one hemisphere removed to relieve intractable epilepsy [15]. In addition the FSP can serve as a predictor of later cognitive and neurological status [19,22]. As the paradigm can be used from an early age on, it can be used to identify risk groups of atypical development early enough to increase the chances of successful intervention. The FSP therefore has a clinical value as a diagnostic tool and surrogate outcome measure.

Previous studies using the FSP have generally relied on measures of saccadic eye movements by a trained, experienced ‘blind’ adult observer judging the time and direction of the infant’s eye movements and making a manual response [2]. The observer controls the onset of the trial, with the stimulus conditions computer-generated in a random sequence to the left or right of the centre. This method has proved effective, but adds the observer’s variable reaction time to the estimate of saccadic latency, making the measures imprecise. Alternatively, saccadic eye movements have been measured by frame-by-frame video analysis [3], which is very time consuming and may be susceptible to human error [23] and has poor spatial resolution [23,24].

Modern eye-trackers can measure infants’ eye-movements with relatively good spatial and temporal accuracy [23,25]. The eye-tracking data can furthermore be processed in real-time to automatically initiate the sequence. This allows a shorter interval between trials, hence the possibility of a larger number of trials before the infant gets tired or bored. The automation furthermore makes it possible to combine the task with other methods that require high temporal accuracy, for example with EEG, to gain insights into the neural mechanisms of attention shifts in infants. As a clinical benefit, automated FSP with eye tracking can be used with minimal prior training of adult observers and possibly to shorter testing times in a clinical context as a diagnostic predictor of disorders of attention which may be present in different developmental disorders.

Eye tracking has been used in similar paradigms in older infants over the age of 4 months to investigate different phenomena, including cueing towards stimuli and emotion processing [e.g. 26,27–29] and it has been used to study interspecies differences in the gap-overlap paradigm between human adults and great apes [30]. The current study investigates the ability to shift attention in a very young age group (aged 1–9 months) by combining eye tracking with the clinically used FSP to accurately measure saccadic latencies under competition and non-competition. Previous comparisons of different stimuli in the FSP indicate that stimulus features are not crucial for the results in regard to attention disengagement [2,3], with the exception of maternal faces which can lead to different saccade latencies [31]. Such stimuli were deliberately avoided in this study and only a schematic face-like stimulus was used here (called here the ‘schematic face’), to be compatible with extensive previous studies from the authors’ group (3, 4, 13, 19–22, 34, 35) and avoid the specific social and affective effects associated with maternal faces. With the increased spatial accuracy of the eye-tracker in comparison to former methods, we also investigate differences in fixation position in relation to the target, such as the undershoot of initial saccades in young infants reported previously [14]. The larger number of trials completed by infants due to the automated approach allowed changes in saccade latency over trials to be investigated, as evidence of learning effects.

## Method

### Participants

The study was approved by the "University College London (UCL) Research Ethics Committee" (Project ID Number: 2002/02, full name of ethics committee: "UCL Research Ethics Committee”). Informed written consent was obtained from the parents or guardians of the infants participating in the study. Two different groups of infants were recruited from a data base of volunteer families. In Group 1, 23 infants, ranging in age from 1.42 to 8.18 months (*M*_*age*_ = 4.19 months, *SD* = 1.74) completed the basic procedure described below. We include data from a further 44 infants between 1.19 and 9.10 months (*M*_*age*_ = 4.59 months, *SD* = 2.38) who completed the same conditions, in the frame of an EEG study that also included other conditions in which targets appeared on both sides of the screen, but was conducted with otherwise identical set up and stimuli. All infants were born within two weeks of full term and were neurologically normal at birth with no perinatal complications. Participating parents received reimbursement of their travel expenses and written informed consent from the parent was obtained on behalf of the infants.

### Materials and Stimuli

**Fig 1:**
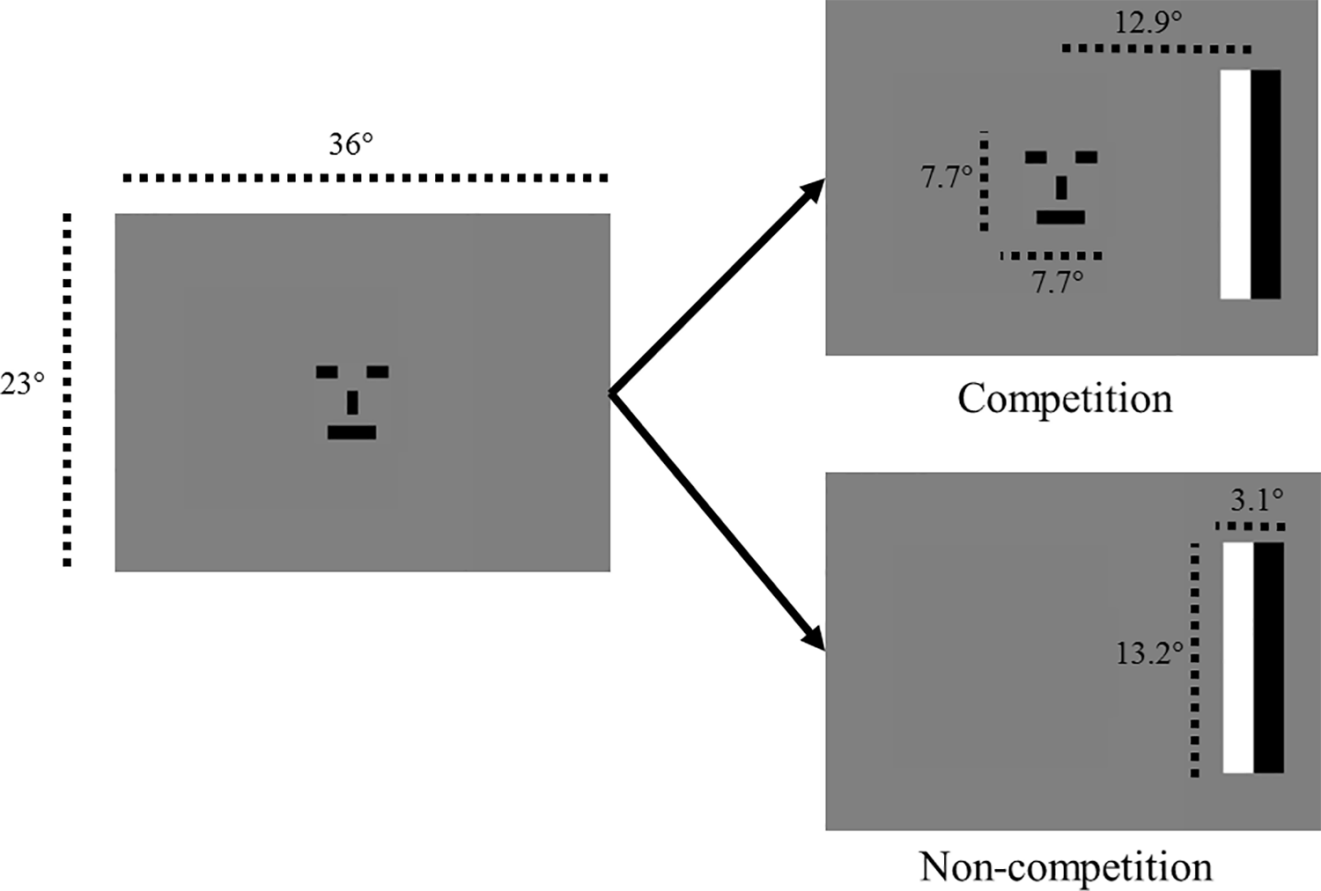
Order of displays shown in competition and non-competition conditions. Stimulus sizes and eccentricities are displayed in degree of visual angle.

The stimuli followed the design as in Hood and Atkinson [3]. The screen and stimulus dimensions are shown in Fig 1. Stimuli appeared on a grey background with a mean luminance of 77 cd/m^2^. Each trial started with an infant-friendly movie being presented for at least 3.3 seconds covering the central 17.5 x 14 degrees of the screen to attract the infant’s attention to the centre of the screen. When the infant was looking at the screen, the first stimulus, the schematic face which switched between two versions at a rate of 3 reversals per second, appeared in the centre of the screen for at least 2 seconds. The distance of the infant’s eyes from the screen was adjusted to obtain the best possible eye-tracker signal which was at approximately 65 cm. When the infant had fixated the face for 0.33 seconds (20 eye tracking samples), the peripherally located stimulus (target) automatically appeared. This threshold was chosen to ensure that the infant fixated on the schematic face at target onset, while not requiring a longer fixation which might increase the infant’s boredom with the test situation. The target consisted of one black and one white rectangular bar that reversed contrast at a rate of 3 reversals per second and appeared to the left or right of the central face stimulus. In the ‘non-competition (NC)’ condition, the central face disappeared at the time when the peripheral target appeared, while in the ‘competition (C)’ condition it remained present throughout the time that the peripheral target was visible on the screen (Fig 1). Stimuli were adapted from Hood and Atkinson [3] to allow for comparability with previous research. The only difference in the stimuli from the original FS paradigm used by Hood and Atkinson [3] was that the eccentricity of the peripheral target was reduced in the current study, due to the screen size and viewing distance (12.9° from centre of screen to centre of the target compared to 22–25° eccentricity in the original paradigm). Stimuli were generated by a DELL PC with Linux operating system, using a MATLAB presentation program presented on an LCD monitor. The relative salience of the central and peripheral stimuli may affect the amount of perceptual processing and therefore saccadic latencies. In the FSP the same stimuli are used in competition and non-competition conditions, controlling for any salience differences. Due to the large supra-threshold stimuli used, the FSP furthermore controls for individual differences in acuity.

### Design

In a mixed design we measured the effect of the within-subject factor Condition (competition or non-competition) and the between-subject factor Age Group on measures of attention switches measured as the latency of an eye-movement towards the target. Sticky fixations on the central schematic face (fixation greater than 5 secs), fixation shift to the ‘wrong’ direction (i.e. the opposite side to the target), ‘corrected’ saccades (an initial fixation shift to the opposite side to where the target had appeared, followed by a corrected saccade in the target direction), and the fixation position after the first saccade towards the target were automatically recorded.

### Eye-tracking

A remote eye-tracker (Tobii X120) was used to sample gaze-position at a rate of 60 Hz. This device, a free-standing unit containing near infrared light sources and cameras uses analysis of the position of the corneal reflections relative to the centre of the illuminated pupils to determine gaze positions of the subject [32]. The eye tracker was controlled via a Matlab program, which was used to extract gaze positions recorded by the eye tracker in real-time during testing. To determine online whether the subject was looking at a stimulus, the distance between the coordinates of the location of the gaze measured by the eye-tracker and the centre of the screen was calculated. If the infant’s fixation was located within the screen coordinates after the infant-friendly movie was visible for a minimum of 3.3 seconds, the central stimulus appeared. The central stimulus remained visible for at least 2 seconds before the target appeared, to avoid the infants becoming upset by the disappearance of the central stimulus as soon as they looked at it. If, after this period, fixation lay within the stimulus for more than 20 samples (0.33 seconds), this was considered a fixation on the stimulus which then triggered the onset of the peripheral target. The central stimulus either disappeared at the onset of the peripheral target (NC = non-competition condition) or remained visible for the duration of the peripheral target (C = competition). The peripheral target disappeared when the infants had fixated it (defined as a fixation within 8.8deg from the centre of the target) for at least 333 ms. S1 Text describes the MATLAB routine which was used to define a correct saccade and to filter out noisy data.

### Procedure

The eye-tracker was calibrated on an adult before the experiment started, using a five point routine, which took no more than two minutes. Pilot work showed that this improved data quality compared to attempting such a calibration procedure on individual infants. In setting-up, it was found with infants aged 3 months and younger that the eye tracker frequently did not initially register a signal from the infants’ pupils. This could usually be overcome (a) by adjusting the infant’s position so that the eye tracker viewed the infant from further below the infant’s line of sight; (b) by placing one or more neutral density filters over the eye tracker face plate to reduce intensity by 0-3-1.5 log units. The success of this method suggests that the youngest infants have a highly reflective fundus of the eye which takes the bright pupil image beyond the dynamic range of the Tobii eye tracker.

The infant was seated on the parent’s lap. Normal room lighting was used throughout testing. The parent wore sunglasses that were opaque to the infra-red used by the eye-tracker to ensure that the eye-tracker did not pick up the parent’s eyes. NC and C conditions, with left- or right-sided targets, were selected by the program in a random order. In the second group of infants these trials were randomly interleaved with trials in different stimulus conditions; therefore this group was not included in the learning effect analyses. Videos were shown between trials to sustain the infants’ attention and if the infants showed any signs of boredom, they were turned away from the screen for a few minutes until they appeared attentive again. Testing continued until the infant became tired or bored, which was usually the case after approximately 10–20 minutes of testing.

## Results

There was no significant difference in saccade latency between the two recruitment groups, *F*(1, 59) = .068, *p* = .796, and no significant interactions of group with the other effects (Age: *F*(1, 59) = 1.197, *p* = .278, Condition: *F*(1, 59) = 0.236, *p* = .627, three-way interaction with Age and Condition: *F*(1, 1941) = 0.110, *p* = .741), suggesting that both subject groups are comparable. Infants from the combined groups were divided into two different age ranges: younger than 4 months (*n* = 30, *M*_age_ = 2.34 months, *SD* = 0.65) and older than 4 months (*n* = 37, *M*_age_ = 6.03 months, *SD* = 1.31), as the main changes in attention shifts have been described to take place between 2 and 4 months from previous studies [review: 16]. Datasets are provided in S1–S3 Datasets.

### Classification of responses

On average infants completed 53.26 trials (*SD* = 20.45, minimum = 24, maximum = 86). Only a small percentage of trials had to be excluded (<12%), including trials when the infant’s fixation was not recorded as being on screen at trial onset, indicating data loss or noise (5%), saccades occurring less than 150 ms after target onset (6%), or trials in which rapid changes in fixation-position were registered, indicating data loss by the eye-tracker (<1%). About 9% of trials showed an initial fixation shift towards the wrong direction. Sticky fixations (no shift within 5 sec) rarely occurred (<2% of trials).

Mixed effects logistic regression analyses with trials nested within subjects were conducted (xtmelogit in Stata 13) to investigate the effects of Condition (NC/C) and Age Group on the number of excluded trials. These occurred significantly more often in the older group (*M* = 14.5%, *STD* = 35.3%) than in younger infants (*M* = 8.6%, *STD* = 28.1%, odds ratio 1.831 (95% CI 1.101–3.046), *z* = 2.32, *p* = .021); there was no effect of Condition on excluded trials (odds ratio 1.074 (95% CI .742–1.553), *z* = 0.38, *p* = .706).

### Latency of refixation

**Fig 2:**
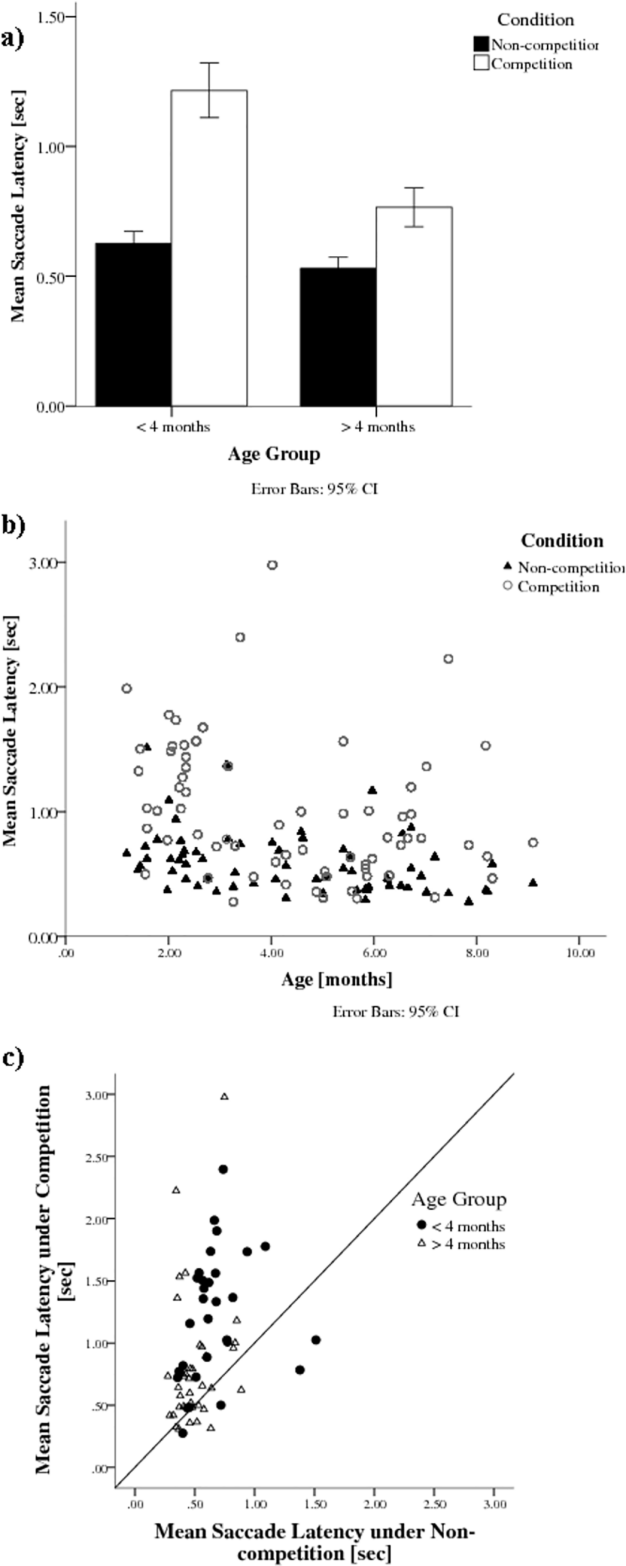
a. Mean time until first saccade for Conditions (non-competition and competition) and Age Groups (younger than 4 months and older than 4 months). b. Mean saccade latencies decrease with age, particularly in the competition condition. c. Mean Saccade Latency in Competition condition compared to Non-competition condition for each individual infant.

Fig 2A and 2B shows the mean latency of correct saccadic shifts from the centre to the peripheral target (refixations) under different conditions for the two age groups (under 4 months and over 4 months). Infants had longer latencies in the competition condition than in the non-competition condition, as highlighted by the individual data points above the 45° line in Fig 2C. A mixed effects linear model revealed significant main effects of both Condition, *F*(1,1944) = 164.13, *p* < .001, *d* = 0.468, with shorter latencies in non-competition (*M* = 572 ms, *STD* = 514) than in competition conditions (*M* = 941 ms, *STD* = 988), and Age, *F*(1,64) = 18.28, *p* < .001, *d* = 0.309, with longer latencies under 4 months of age (*M* = 895 ms, *STD* = 840) than over 4 months (*M* = 648 ms, *STD* = 756), as well as a significant interaction of Condition with Age, *F*(1, 1944) = 27.55, *p* < .001.

### Variation of latencies over trials

To investigate changes in saccade latencies over trial number, hierarchical linear models with mixed effects were calculated. The fit of the model was improved by including a variable slope, compared to a fixed slope, as well as an intercept (2 * Difference in log-likelihood = 6.64). The slope was negative but not significant (95% CI -.0189 - .0014, *z* = -1.69, *p* = 0.091). Including a quadratic term did not improve the predictive value of the model.

**Fig 3:**
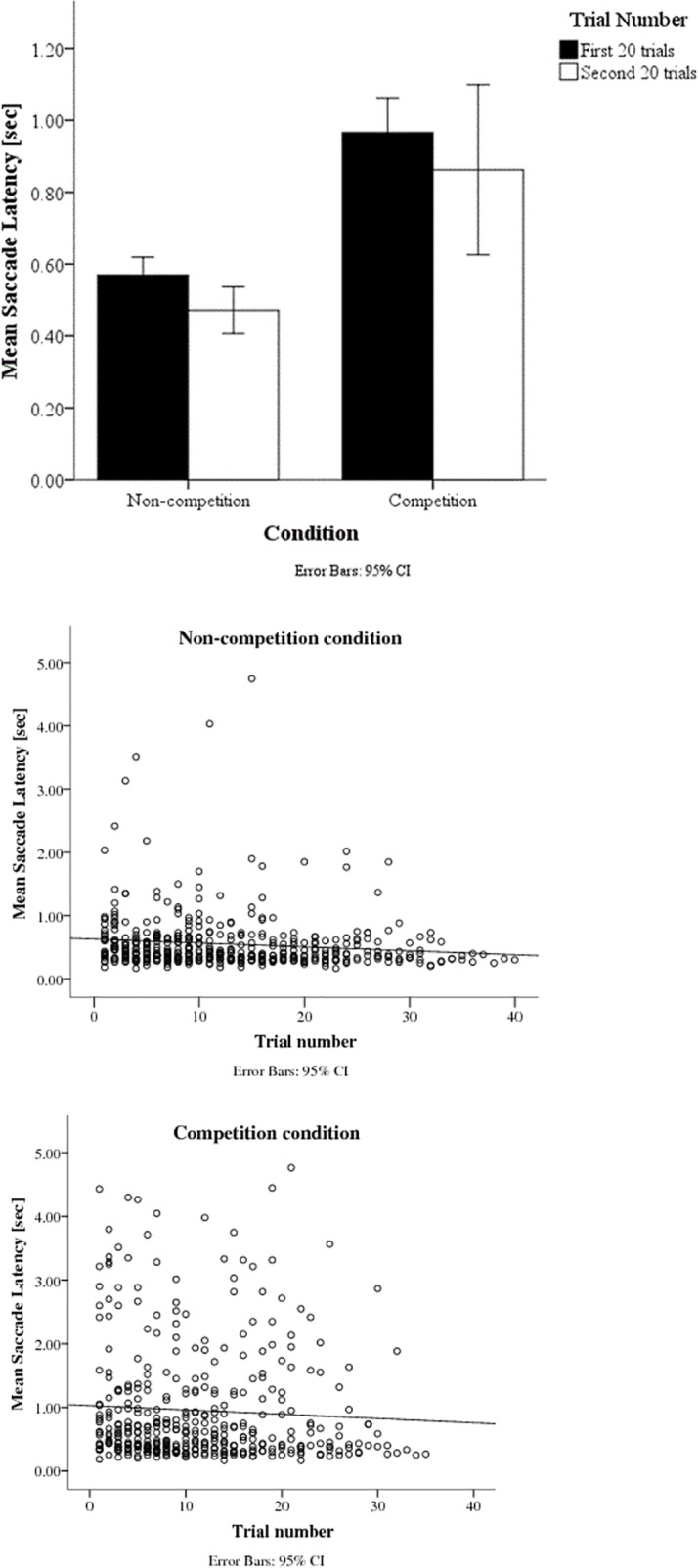
a. Saccade latency in the first 20 trials and the second 20 trials separately for each condition. Saccade latencies are generally shorter in the second 20 trials. Competition latencies show higher variance which may be the reasons for the absence of a significant effect of trial number in this condition. b. Scatter plot of individual infants’ latencies as a function of trial number in the non-competition condition. The slope is significantly negative (p = .011). The reduction in very long latencies across trials may be the major contributor to the learning effect. c. Scatter plot of individual infants’ latencies as a function of trial number in the competition condition. The slope did not significantly differ from zero (p = .929), suggesting that there is no learning effect under competition

When calculating separate models for competition and non-competition conditions, the trial number was not a significant predictor in the competition condition, 95% CI -.016 - .018, *z* = 0.09, *p* = 0.929, but there was a significantly negative slope in the non-competition condition, 95% CI -.011–-.001, *z* = -2.55 *p* = 0.011, Fig 3.

### Sticky fixations

**Fig 4:**
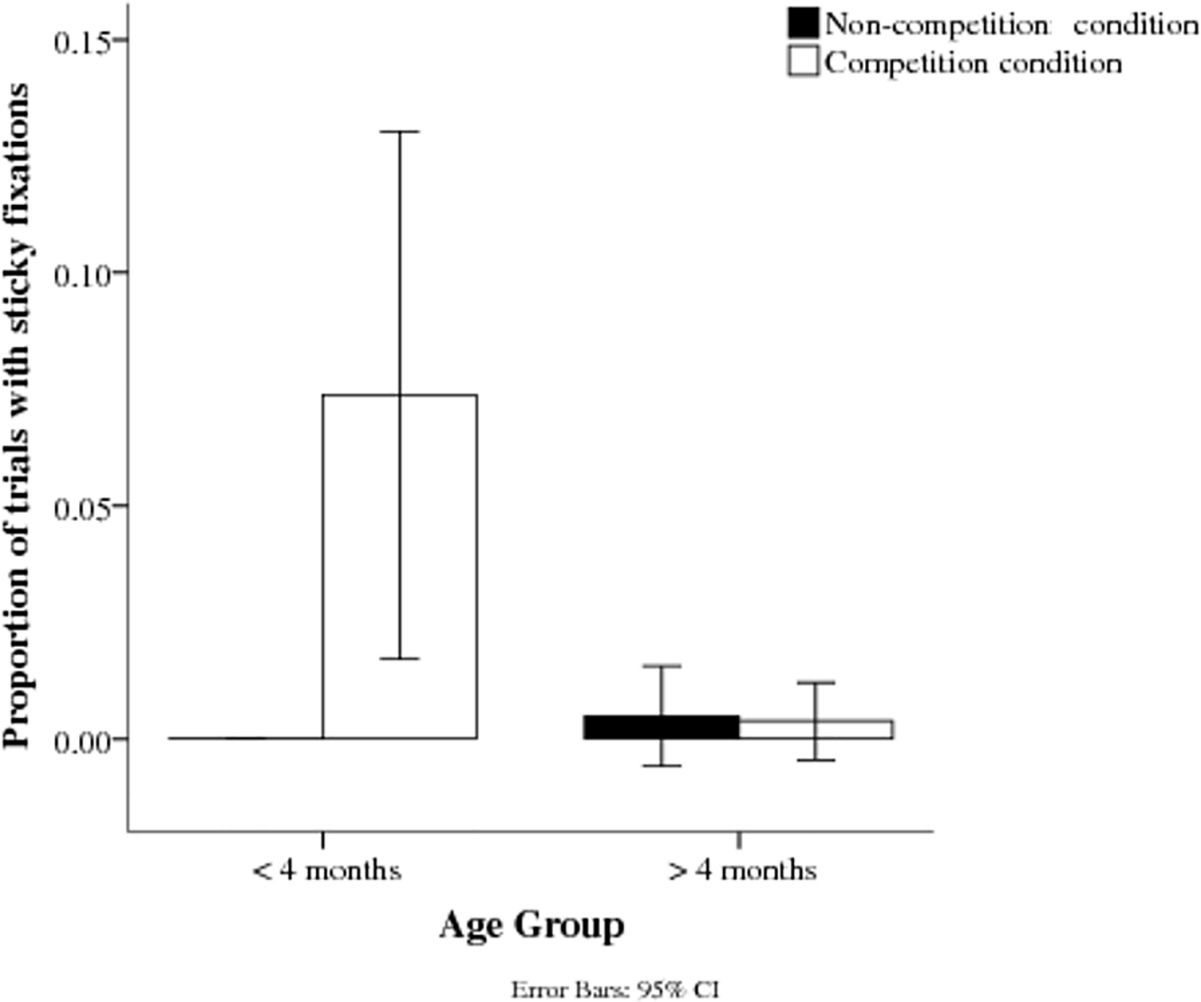
Proportion of fixations stuck on the central stimulus for the two age groups in non-competition and competition conditions.

The proportion of sticky fixations was smaller in the non-competition condition than in the competition condition (Fig 4). This difference was significant in a mixed effects logistic regressions performed in R (version 0.97.551, RStudio, Inc., 2009–2012), *z* = 6.816, *p* < 0.001. Although the main effect of age was not significant, *z* = -1.934, *p* = .053, a significant Age x Competition interaction, *z* = -2.458, *p* = .014, suggests that the effect was particular marked for competition in the younger group.

### Extent of saccadic shift

**Fig 5:**
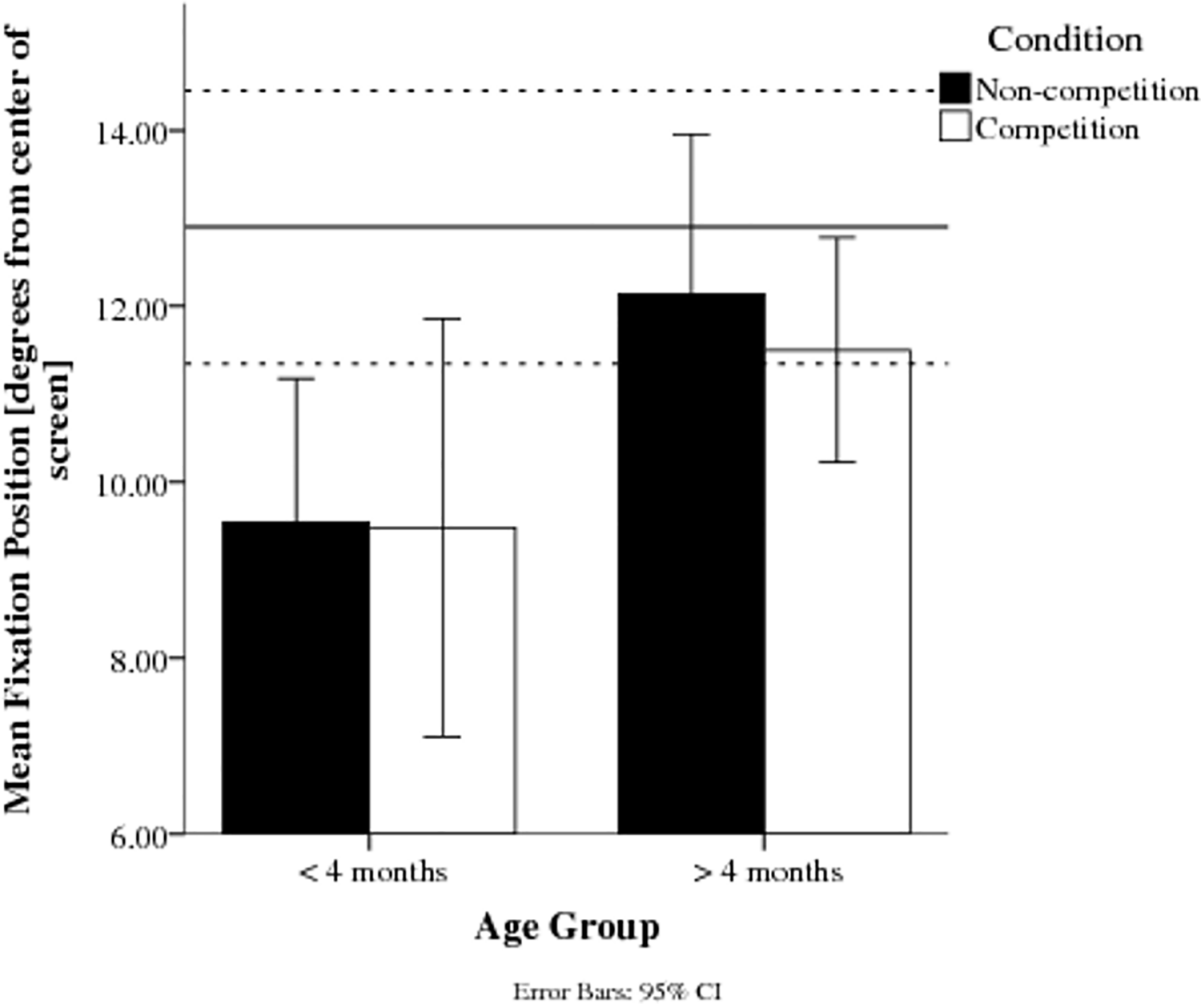
Average fixation position from the centre of the screen in degrees of visual angle after the first saccade towards the target. The dashed lines signify the target position. Younger infants’ fixation position is closer to the centre of the screen than the older infants’ in both conditions.

At the end of the first saccade towards the target, younger infants’ fixations were closer to the centre of the screen; i.e. the saccade undershot the peripherally located target (Fig 5). A general linear model for repeated measures confirmed this difference was significant between Age Groups, *F*(1,21) = 4.72, *p* < .05, *d* = 0.833, but not between Conditions, *F*(1,21) = 0.63, *p* = .438; with no significant Condition x Age Group interaction, *F*(1,21) = 0.42, *p* = .522. Older infants’ initial fixations were within the region of the target (on average at an eccentricity of 11.8°, *SD* = 2.5), whereas the younger infants did not reach the target (average eccentricity of saccade: 9.5°, *SD* = 3.0). As the target disappeared after the first saccade towards it, subsequent corrections of the undershoot could not be further investigated as these further eye movements were not recorded.

## Discussion

Saccade latencies were longer in the competition condition than in the non-competition condition, and this effect was greater in the younger group of infants under 4 months of age, supporting the findings of former studies using this paradigm [2,3]. Saccade latencies decreased over trial number, suggesting a small learning effect in the non-competition condition, but this was not seen in the competition condition. Sticky fixations mostly occurred in younger infants under competition. Furthermore, younger infants’ saccades initially undershot the target position, in agreement with Aslin and Salapatek [14].

### Classification of responses

Infants completed successfully a large number of trials that is comparable to, or exceeds, that in former studies [20 trials: 2,27 to 37 trials: 3], even after trial exclusions, confirming the expectation that eye-tracking can increase trial numbers. The small number of excluded trials were mostly due to fixation away from the screen centre at trial onset, indicating signal loss, or the saccade being too early to be a response to the target. Less than 1% of trials were lost as a result of excessive eye-tracking noise. Overall, the data quality indicates that, in these age groups, eye-tracking can be used to investigate visual attention with a success rate at least as good as previous testing methods.

### Latency of refixation

The longer latency under competition confirms our prediction that infants, particularly under 4 months, have problems disengaging attention when the central target remains visible. These findings are in line with other studies using the Fixation Shift Paradigm [2,3]. Given that the FSP can be used as a predictor of developmental problems and a diagnostic tool to identify high-risk groups for attention problems [review: 4,15,18,19], eye-tracking should improve its usability, as it avoids the need for experienced observers, and provides more trials in a short testing time, both of which are likely to be important in a clinical context.

Further, eye-tracking data can be analysed by the computer while the experiment is still running, and can be processed and fed back into the stimulus presentation programme, allowing combination of behavioural measures with other methods that need accurate registration of saccadic timing, for example electroencephalography (EEG) and other neuroimaging methods. Our behavioural results show that additional processing time is required under competition, which is in line with the previous idea that the developmental improvement in competition performance requires time-consuming cortical control over reflex-like subcortical attention [13,15,33], but do not provide direct evidence on the brain networks involved in fixation shifts and disengagement. The possibility to combine the Fixation Shift Paradigm with EEG, exploiting the improved temporal precision of eye-tracking, makes it possible to study the time course of target-induced brain responses in different brain areas and in linked networks, giving insights into how the cortical control of attention develops. These comparisons, using simultaneous recording of saccadic latencies with EEG recordings of brain activity related to shifts of attention are underway at present in our laboratory [34,35].

### Variation of latencies over trials

Independent of age, infants became quicker at shifting attention the more trials they completed, suggesting a learning effect. The short cartoon clips between trials may have acted as a reward, reinforcing prompt fixation shifts that make the cartoons reappear earlier. However, no such learning effect was found for the slower and more variable responses that occur in competition conditions. This suggests that this type of learning effect may not influence the ability to disengage from an existing stimulus, but only processes that are involved in shifting attention to a new target. Alternatively, the variability of saccadic latencies in the competition condition compared to the non-competition condition and individual differences across infants in saccadic latencies may lead to non-significant effects in the competition condition. It would be interesting to investigate whether there is a long lasting and generalised improvement in infants’ saccadic shifts, or whether it occurs only in the specific testing situation.

### Sticky fixations

“Sticky fixations” only rarely occurred, confirming that the latency of refixation should not be distorted in this age group by data in which infants failed to shift within the defined time after target onset. The low number of sticky fixations is in line with former studies showing that infants rarely fail to shift gaze after an age of two months [3,9,13]. Furthermore, the target bars were closer to the centre of the screen than in former studies [3], possibly making it easier for infants to shift towards them. However, sticky fixations occurred more often in the competition condition and in younger infants, an effect that is comparable to results of former studies. A larger group of very young infants (under 2 months) might well have shown a higher incidence of sticky fixations.

### Gaze position

Our results indicate that younger infants’ initial fixations land closer to the centre of the screen, consistent with the finding that young infants tend to initially undershoot the target [14]. As competition and non-competition conditions of the FSP did not affect the landing point of the initial saccade, difficulty in disengaging attention does not seem to be responsible for these inaccurate saccades. Our results therefore suggest that the motor programming or execution of eye-movements is itself immature in younger infants, in addition to the attentional control of disengagement.

## Conclusion

Eye-tracking provides good temporal resolution, providing more information about timing than previous direct observation methods. The sampling rate of 60 Hz in this study allows measurement of the saccade latencies to within +/-8.33 ms, as well as a detailed inspection of different steps during the disengagement process. This increased accuracy may have been crucial for uncovering the learning effects over trial number. Furthermore, the greater spatial accuracy made it possible to investigate fixation positions, which cannot accurately be judged by adult observers or video coding. The automation leads to shorter testing times, making this approach suitable as a quick test in clinical contexts. In general, eye-tracking can increase accuracy and lead to higher trial numbers, suggesting that this methodology provides a significant improvement for studying the development of attention and opportunities to detect further behavioural phenomena in attention tasks.

Use of the eye tracker with this FSP method allows for the possibility of a completely automated efficient clinical tool for the diagnosis of early development of attention shifts, monitored through eye movements. Our results confirm the importance of the ability to disengage for normal development of attention in the first few months of life. The increase in accuracy and trial number enabled identification of a new learning effect, with improving saccadic latencies over time. The data also supported evidence of an initial undershoot of the target by younger infants, suggesting that oculomotor as well as attentional components of the task improve over the age range tested. In conclusion, eye-tracking can efficiently give precise insights into attention development, improving the efficiency of using the FSP as an early diagnostic tool of attention deficits in early infancy and an early surrogate outcome measure and offers the possibility of using on-line data linked to EEG recording.

## Supporting Information

S1 Text (PDF)

S1 Dataset (XLSX)

S2 Dataset (XLSX)

S3 Dataset (XLSX)
