# Supplementary material for: Automatic Detection of Attention Shifts in Infancy: Eye Tracking in the Fixation Shift Paradigm
Source: PLoS One. 2015 Dec 1;10(12):e0142505. doi: 10.1371/journal.pone.0142505 (PMC4666405; doi:10.1371/journal.pone.0142505)
Supplement: S1 Text — (PDF) [file pone.0142505.s001.pdf]

S1 Text

Algorithm to interpolate and analyse eye-tracking data

If eye-position data was missing in a sample, the data in this sample was interpolated with the average of the previous sample the first subsequent successful sample. A saccade was defined as a horizontal change of gaze-position on the screen by more than 2.2 degree of visual angle between two successive samples.

Noisy trials were excluded according to the following criteria: (1) if the gaze position at the onset of the second stimulus (target) was not within 900 pixel horizontal or 500 pixel vertical from the centre of the screen, indicating that the eye tracker lost signal at target onset, (2) if the trial contained too many excursions in fixation position (> 20% of samples showed excursions greater than 2.2 degree of visual angle compared to the previous sample) indicating fuzziness or signal-loss from the eye-tracker, or (3) if the first saccade occurred earlier than 0.15 seconds after the appearance of the peripheral target, as it is very unlikely that those saccades were related to the appearance of the target.

Trials in which the first saccade occurred later than 5 seconds after target onset were registered as a sticky fixation and excluded from the analysis of latencies. Trials with the initial saccade to the wrong direction were excluded from the analysis. For non-noisy trials with the first saccade towards the correct direction, the latency (difference in time between target onset and first lateral saccade towards it) was calculated and averaged within each condition. The gaze position (in degree of visual angle from the centre of the screen) after the first saccade was calculated by averaging across trials after the first saccade towards the correct direction either until the next saccade occurred away from the fixation position or for a maximum of 10 samples.
